# Supplementary material for: Self-organized and self-sustained ensemble activity patterns in simulation of mouse primary motor cortex
Source: bioRxiv. 2025 Jan 14:2025.01.13.632866. Preprint. [Version 1] doi: 10.1101/2025.01.13.632866 (PMC11760730; doi:10.1101/2025.01.13.632866)
Supplement: 1 [file NIHPP2025.01.13.632866V1-supplement-1.pdf]

bioRxiv preprint doi: https://doi.org/10.1101/2025.01.13.632866; this version posted January 14, 2025. The copyright holder for this preprint (which was not certified by peer review) is the author/funder, who has granted bioRxiv a license to display the preprint in perpetuity. It is made available under aCC-BY 4.0 International license.

Supplementary Data

Self-organized and self-sustained ensemble activity patterns in simulation of mouse primary motor cortex

Doherty, D.W.[1], Jung, J.[1], Dura-Bernal[1,2], and Lytton, W.W.[1,3]

[1] Department of Physiology & Pharmacology, SUNY Downstate Health Sciences University, Brooklyn, NY 11203, USA, [2] Nathan Kline Institute for Psychiatric Research, Orangeburg, NY, USA, [3] Kings County Hospital, Brooklyn, NY 11203, USA.

bioRxiv preprint doi: https://doi.org/10.1101/2025.01.13.632866; this version posted January 14, 2025. The copyright holder for this preprint (which was not certified by peer review) is the author/funder, who has granted bioRxiv a license to display the preprint in perpetuity. It is made available under aCC-BY 4.0 International license.

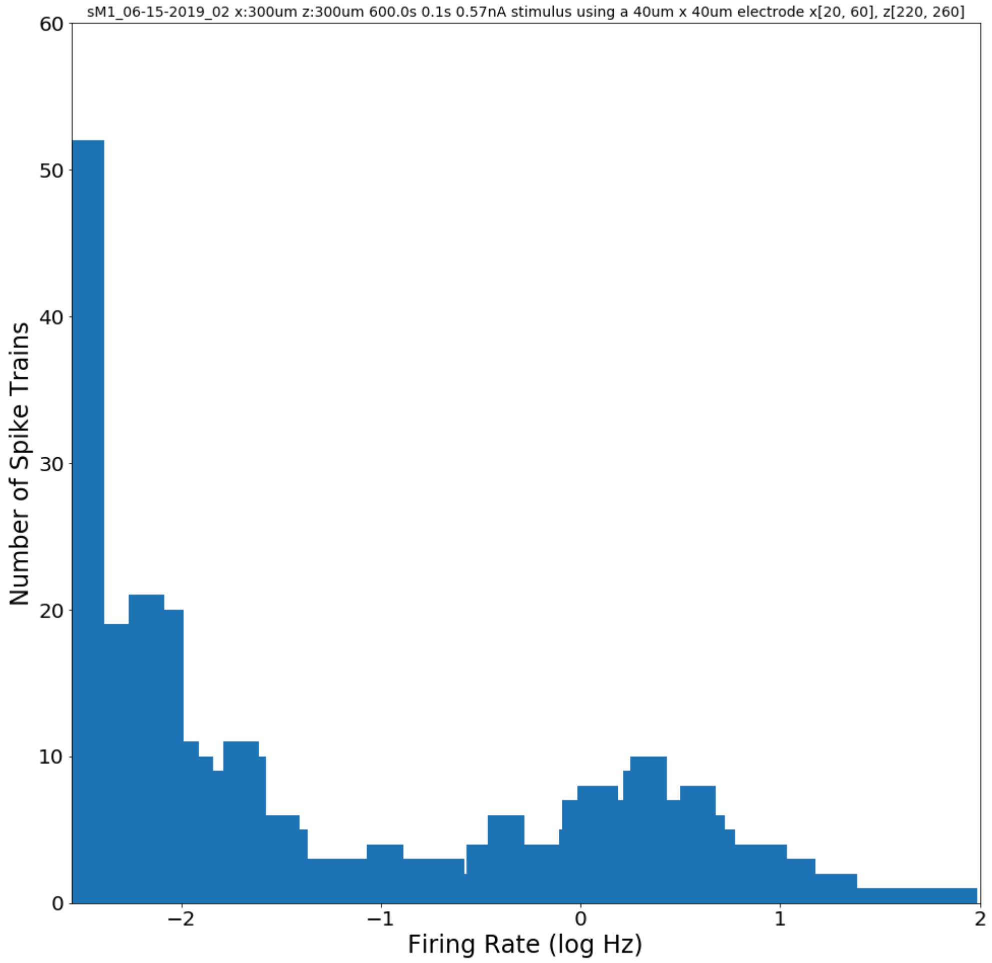

Figure S1. The number of spike trains with logarithm of firing rate (Hertz) for the entire population of neurons. The right hand side shows the lognormal distribution of firing rates typical of firing rate data collected in vivo (from about -0.5 log or 0.32 Hz to about 1.8 log or 63 Hz). At left are very low firing rates (from about -2.5 log or 0.003 Hz to about -1.3 log or 0.05 Hz) that form a power curve distribution. Simulation sM1_06-15-2019_02. Row 2 in Fig S2.

bioRxiv preprint doi: https://doi.org/10.1101/2025.01.13.632866; this version posted January 14, 2025. The copyright holder for this preprint (which was not certified by peer review) is the author/funder, who has granted bioRxiv a license to display the preprint in perpetuity. It is made available under aCC-BY 4.0 International license.

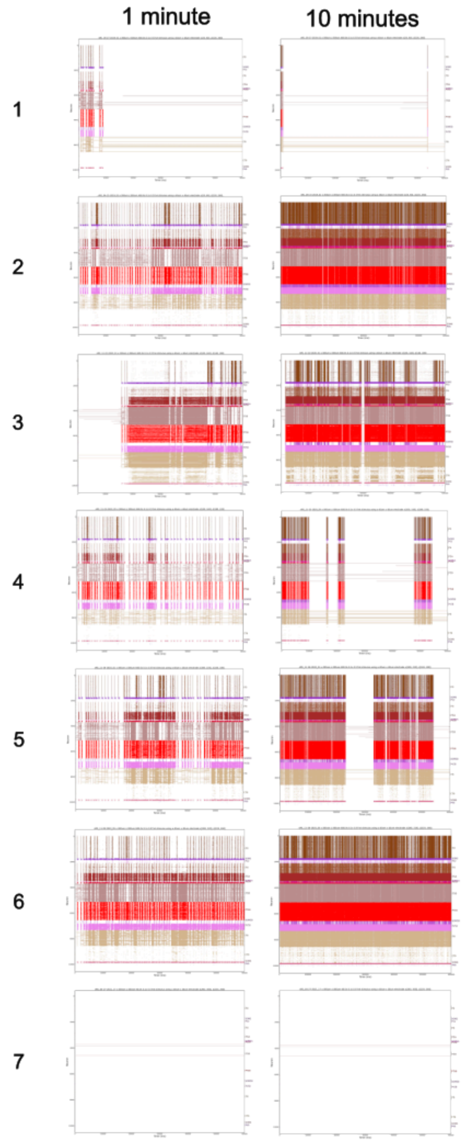

Figure S2. Seven simulations of 10 min cortical activity, each with different connectivities (different connectivity each row). Left column shows raster plots of all neurons for 1 min. Right column shows raster plots of all neurons for 10 min. Row 1: sM1_05-27-2019_01, 2: sM1_06-15-2019_02, 3: sM1_11-22-2020_01, 4: sM1_11-23-2020_05, 5: sM1_11-26-2020_01, 6: sM1_11-28-2020_05, 7: sM1_04-27-2021_17.

bioRxiv preprint doi: https://doi.org/10.1101/2025.01.13.632866; this version posted January 14, 2025. The copyright holder for this preprint (which was not certified by peer review) is the author/funder, who has granted bioRxiv a license to display the preprint in perpetuity. It is made available under aCC-BY 4.0 International license.

Row	Simulation	Seed	Duration (ms)	Avalanches
1	sM1_05-27-2019_01	cfg.seeds = {'conn': 4321, 'stim': 1234, 'loc': 4321}	600,000	93,020
2	sM1_06-15-2019_02	cfg.seeds = {'conn': 5294, 'stim': 3812, 'loc': 6017}	600,000	15,578
3	sM1_11-22-2020_01	cfg.seeds = {'conn': 3830, 'stim': 3084, 'loc': 3091}	600,000	9,750
4	sM1_11-23-2020_05	cfg.seeds = {'conn': 9364, 'stim': 7334, 'loc': 3329}	600,000	102,739
5	sM1_11-26-2020_01	cfg.seeds = {'conn': 2484, 'stim': 9931, 'loc': 8516}	600,000	38,306
6	sM1_11-28-2020_05	cfg.seeds = {'conn': 4224, 'stim': 4095, 'loc': 7058}	600,000	2,039
7	sM1_04-27-2021_17	cfg.seeds = {'conn': 3209, 'stim': 3639, 'loc': 4626}	600,000	32,969

Table S1. Seven simulations from Fig S2 with values under Row matching the row labels in Fig S2. Simulation: simulation name. Seed: random number seeds provided for ‘conn’ (connections), ‘stim’ (stimulus), and ‘loc’ (locations). Duration (ms): simulation duration in milliseconds. Avalanches: total number of avalanches across the full duration of the simulation.

Avalanche Type	Number	Percent Total Num	Size Range (spikes)	Size Range (neurons)	Duration Range (ms)	Total Duration (ms)	Percent Total Dur
Irregular	92,844	99.81%	1-55	1-55	1-21	126,303	95.0%
Fragment	166	0.18%	1-434	1-281	1-94	1,165	1.0%
Beta							
Delta+	10	0.01%	5,934-61,194	1,585-7,424	220-1,372	5,364	4.0%
Total	93,020	100.0%				132,832	100.0%

Table S2. Data from a 10 minutes simulation of M1 (sM1_05-27-2019_01). Row 1 in Fig S2.

bioRxiv preprint doi: https://doi.org/10.1101/2025.01.13.632866; this version posted January 14, 2025. The copyright holder for this preprint (which was not certified by peer review) is the author/funder, who has granted bioRxiv a license to display the preprint in perpetuity. It is made available under aCC-BY 4.0 International license.

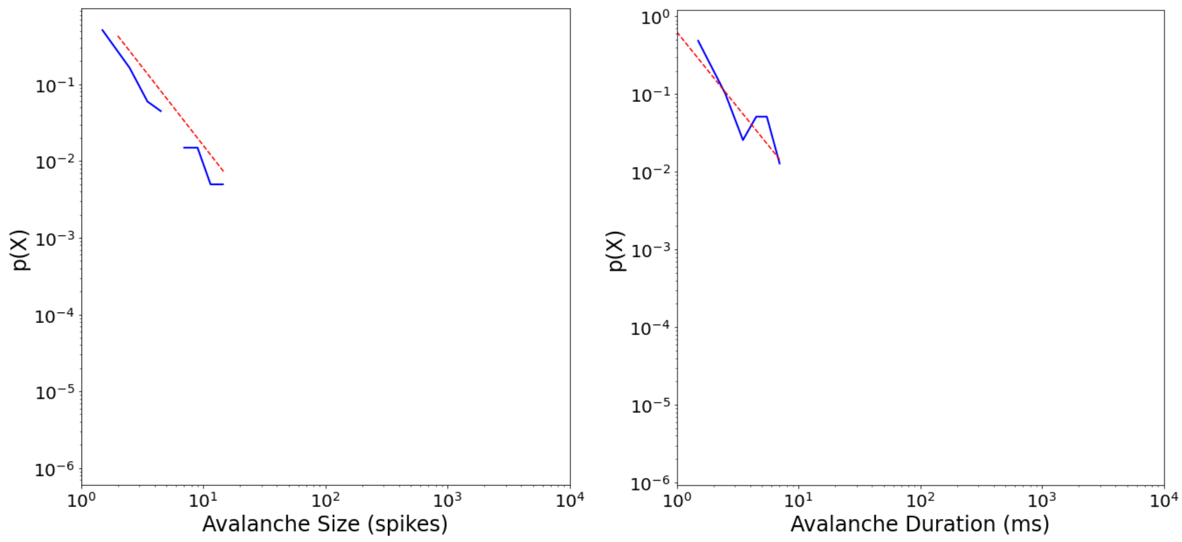

Figure S3. Power-law fits for avalanche size to all neurons in our 10 minute simulation of an M1 cortical column (sM1_05-27-2019_01). Left: Avalanche size probability density distribution with the number of avalanches normalized (y-axis) and the size of the avalanche (number of spikes; x-axis). Power-law fit equals -2.04 (red dashed line; sigma = 0.21, D = 0.085). Right: Avalanche duration probability density distribution showing the number of avalanches normalized (y-axis) and their durations (milliseconds; x-axis). Power-law fit equals -1.94 (red dashed line; sigma = 0.18, D = 0.055). Analyzed using the Python powerlaw package [44].

bioRxiv preprint doi: https://doi.org/10.1101/2025.01.13.632866; this version posted January 14, 2025. The copyright holder for this preprint (which was not certified by peer review) is the author/funder, who has granted bioRxiv a license to display the preprint in perpetuity. It is made available under aCC-BY 4.0 International license.

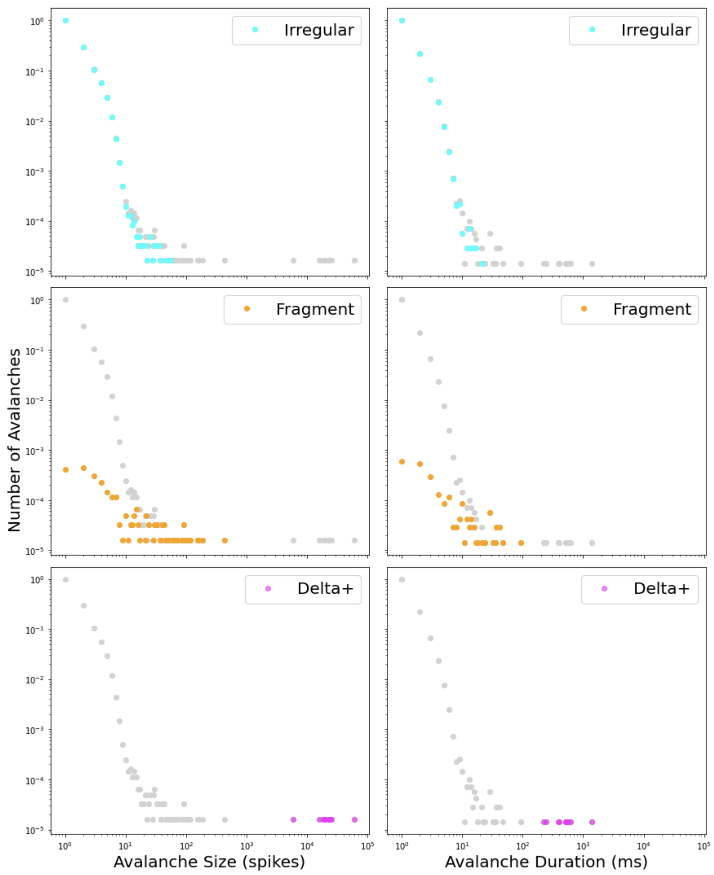

Figure S4. Log-log probability density distributions of avalanche size (first column) and duration (second column) for all avalanches (gray) and each of the three avalanche types seen in this simulation (sM1_05-27-2019_01; row 1; Fig S2).

bioRxiv preprint doi: https://doi.org/10.1101/2025.01.13.632866; this version posted January 14, 2025. The copyright holder for this preprint (which was not certified by peer review) is the author/funder, who has granted bioRxiv a license to display the preprint in perpetuity. It is made available under aCC-BY 4.0 International license.

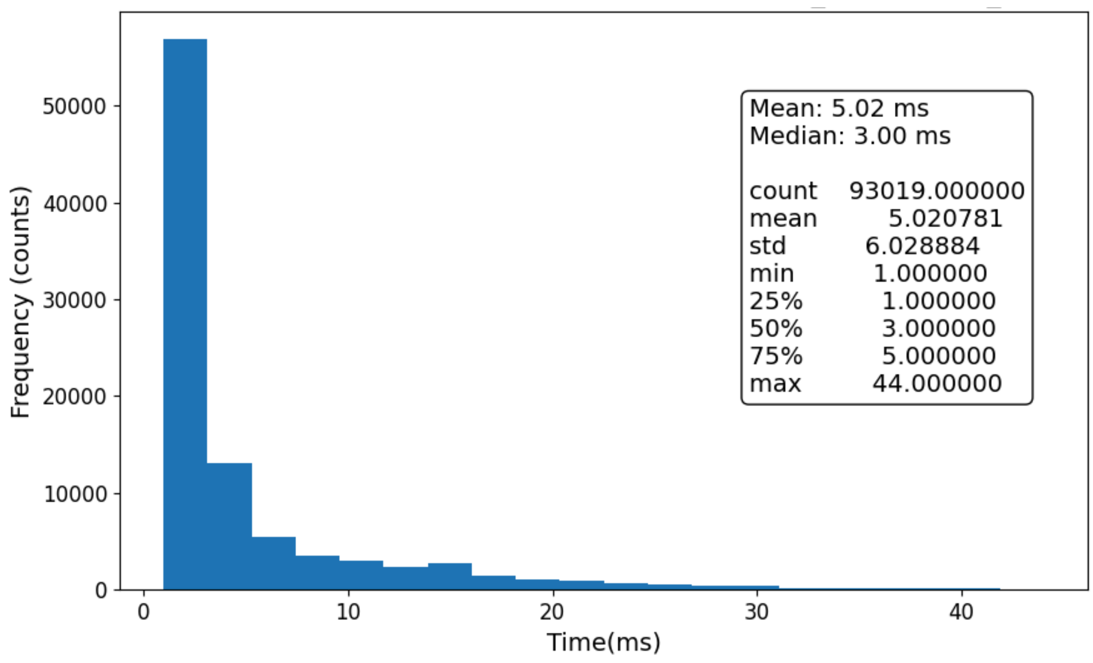

Figure S5. Inter-avalanche intervals in milliseconds (sM1_05-27-2019_01; row 1; Fig S2).

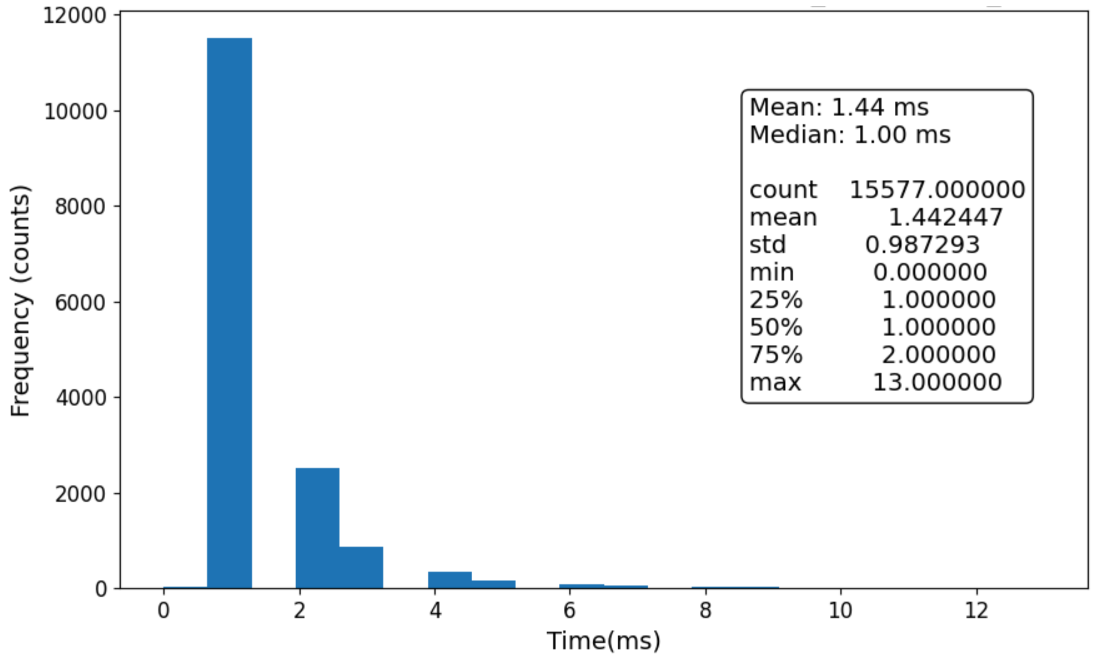

Figure S6. Inter-avalanche intervals in milliseconds (sM1_06-15-2019_02; row 2; Fig S2).

bioRxiv preprint doi: https://doi.org/10.1101/2025.01.13.632866; this version posted January 14, 2025. The copyright holder for this preprint (which was not certified by peer review) is the author/funder, who has granted bioRxiv a license to display the preprint in perpetuity. It is made available under aCC-BY 4.0 International license.

Avalanche Type	Number	Percent Total Num	Size Range (spikes)	Size Range (neurons)	Duration Range (ms)	Total Duration (ms)	Percent Total Dur
Irregular	8,201	84.1%	1-76	1-75	1-32	16,941	3.0%
Fragment	1,366	14.0%	1-2,034	1-2,022	1-249	18,709	3.3%
Beta	34	0.4%	925-153,157	886-4,311	34-4,646	8,129	1.4%
Delta+	149	1.5%	4,045-2,465,599	1,265-7,868	124-67,872	524,817	92.3%
Total	9,750	!00.0%				568,596	100.0%

Table S3. Data from a 10 minutes simulation of M1 (sM1_11–22-2020_01). Row 3 in Fig S2.

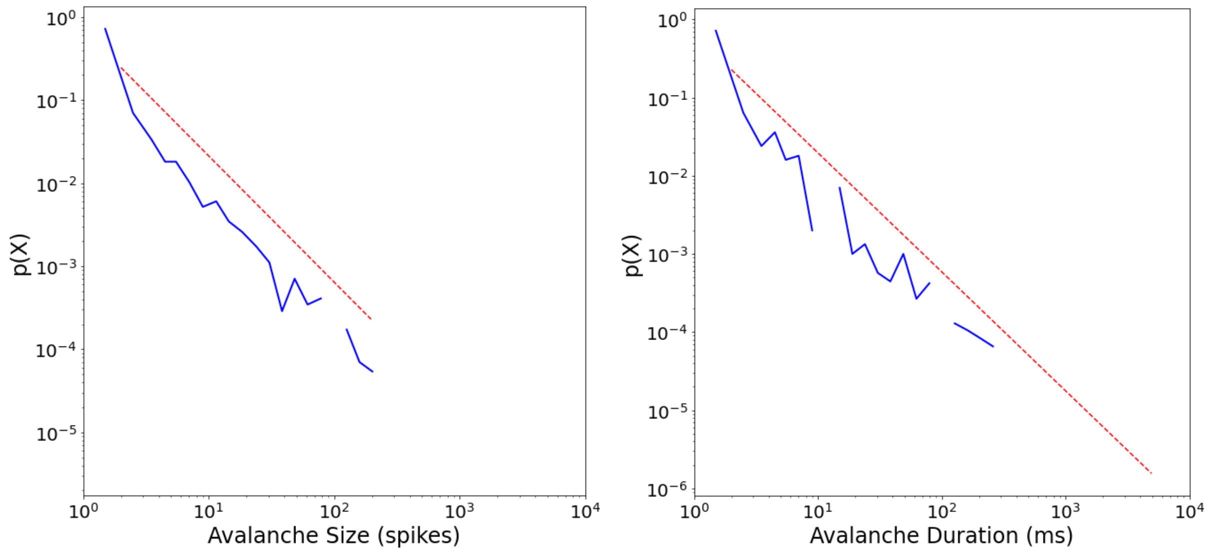

Figure S7. Power-law fits for avalanche size to all neurons in our 10 minute simulation of an M1 cortical column (sM1_11–22-2020_01). Left: Avalanche size probability density distribution with the number of avalanches normalized (y-axis) and the size of the avalanche (number of spikes; x-axis). Power-law fit equals -1.52 (red dashed line; sigma = 0.052, D = 0.023). Right: Avalanche duration probability density distribution showing the number of avalanches normalized (y-axis) and their durations (milliseconds; x-axis). Power-law fit equals -1.50 (red dashed line; sigma = 0.061, D = 0.038). Analyzed using the Python powerlaw package [44].

bioRxiv preprint doi: https://doi.org/10.1101/2025.01.13.632866; this version posted January 14, 2025. The copyright holder for this preprint (which was not certified by peer review) is the author/funder, who has granted bioRxiv a license to display the preprint in perpetuity. It is made available under aCC-BY 4.0 International license.

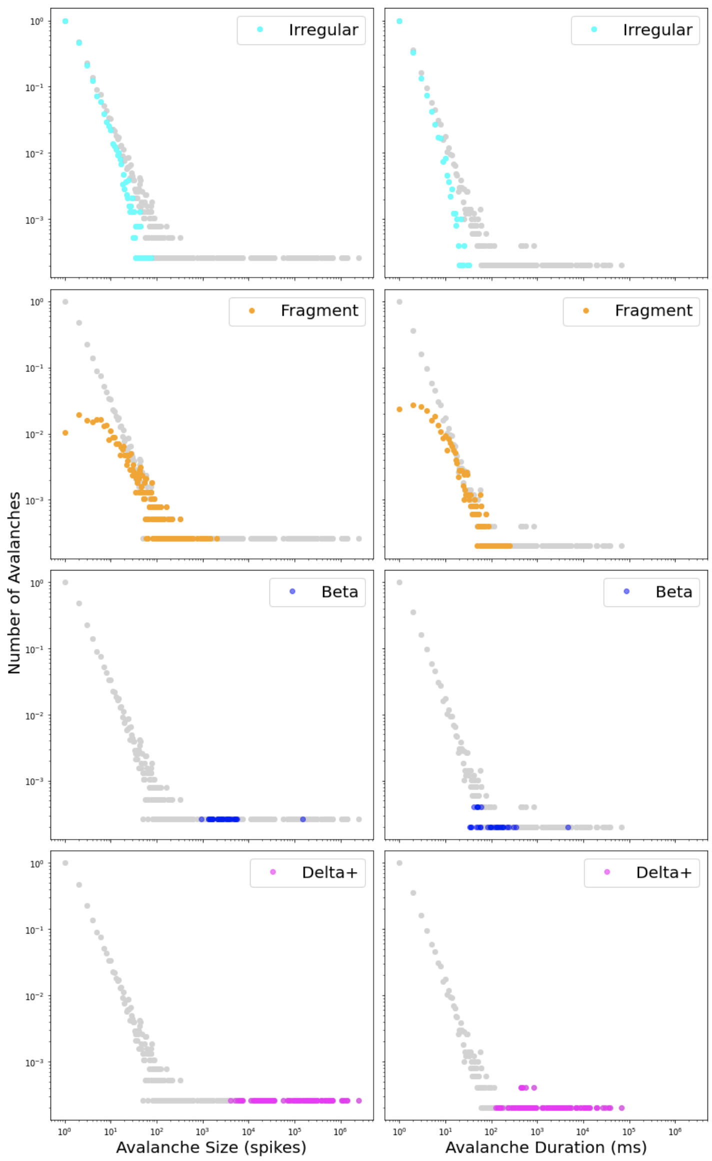

Figure S8. Log-log probability density distributions of avalanche size (first column) and duration (second column) for all avalanches (gray) and each of four avalanche types (sM1_11–22-2020_01; row 3; Fig S2).

bioRxiv preprint doi: https://doi.org/10.1101/2025.01.13.632866; this version posted January 14, 2025. The copyright holder for this preprint (which was not certified by peer review) is the author/funder, who has granted bioRxiv a license to display the preprint in perpetuity. It is made available under aCC-BY 4.0 International license.

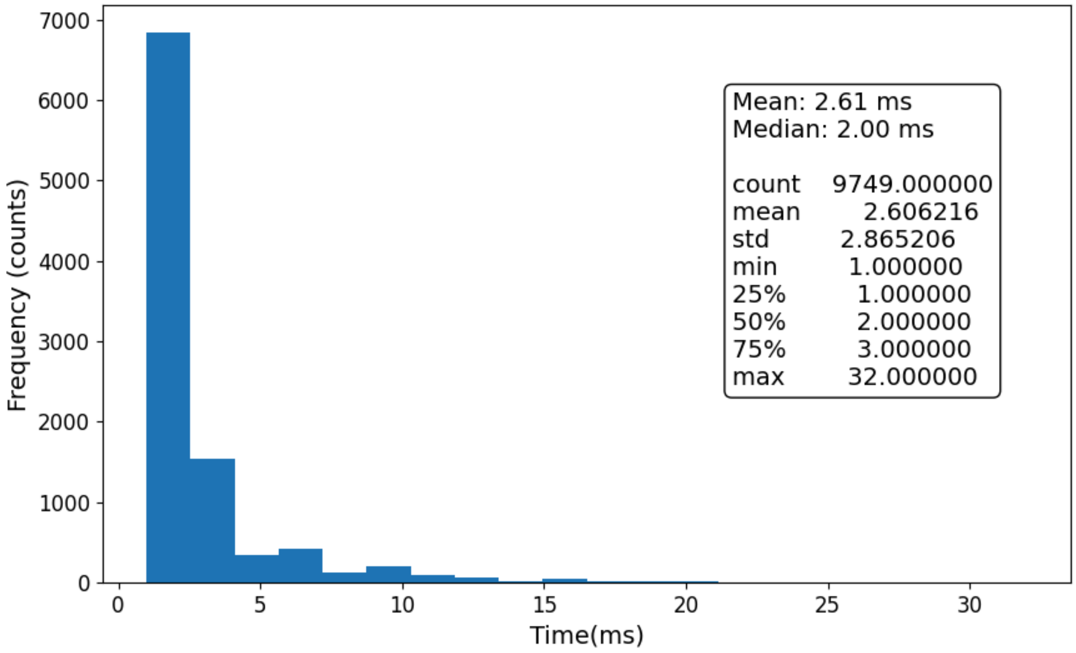

Figure S9. Inter-avalanche intervals in milliseconds (sM1_11–22-2020_01; row 3; Fig S2).

Avalanche Type	Number	Percent Total Num	Size Range (spikes)	Size Range (neurons)	Duration Range (ms)	Total Duration (ms)	Percent Total Dur
Irregular	99,448	96.80%	1-126	1-126	1-42	167,920	58.4%
Fragment	3,097	3.01%	1-1,677	1-609	1-246	26,855	9.3%
Beta	13	0.01%	933-6,622	867-5,012	38-268	1,013	0.4%
Delta+	182	0.18%	1,557-197,405	1,058-7,569	31-3,293	91,784	31.9%
Total	102,739	100.0%				287,572	100.0%

Table S4. Data from a 10 minutes simulation of M1 (sM1_11-23-2020_05). Row 4 in Fig S2.

bioRxiv preprint doi: https://doi.org/10.1101/2025.01.13.632866; this version posted January 14, 2025. The copyright holder for this preprint (which was not certified by peer review) is the author/funder, who has granted bioRxiv a license to display the preprint in perpetuity. It is made available under aCC-BY 4.0 International license.

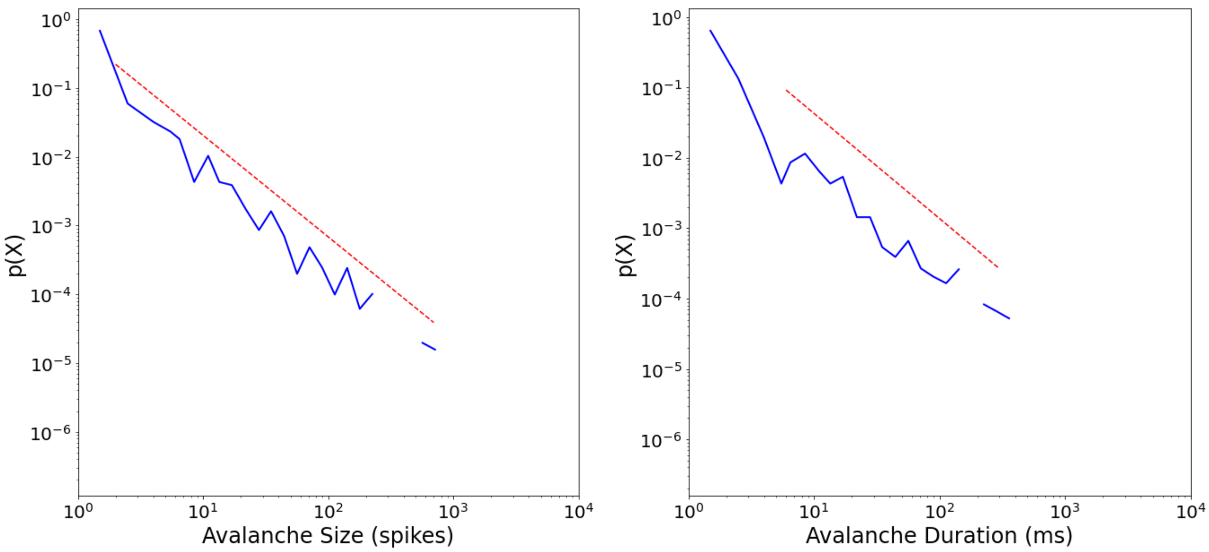

Figure S10. Power-law fits for avalanche size to all neurons in our 10 minute simulation of an M1 cortical column (sM1_11-23-2020_05). Left: Avalanche size probability density distribution with the number of avalanches normalized (y-axis) and the size of the avalanche (number of spikes; x-axis). Power-law fit equals -1.48 (red dashed line; sigma = 0.044, D = 0.028). Right: Avalanche duration probability density distribution showing the number of avalanches normalized (y-axis) and their durations (milliseconds; x-axis). Power-law fit equals -1.49 (red dashed line; sigma = 0.082, D = 0.057). Analyzed using the Python powerlaw package [44].

bioRxiv preprint doi: https://doi.org/10.1101/2025.01.13.632866; this version posted January 14, 2025. The copyright holder for this preprint (which was not certified by peer review) is the author/funder, who has granted bioRxiv a license to display the preprint in perpetuity. It is made available under aCC-BY 4.0 International license.

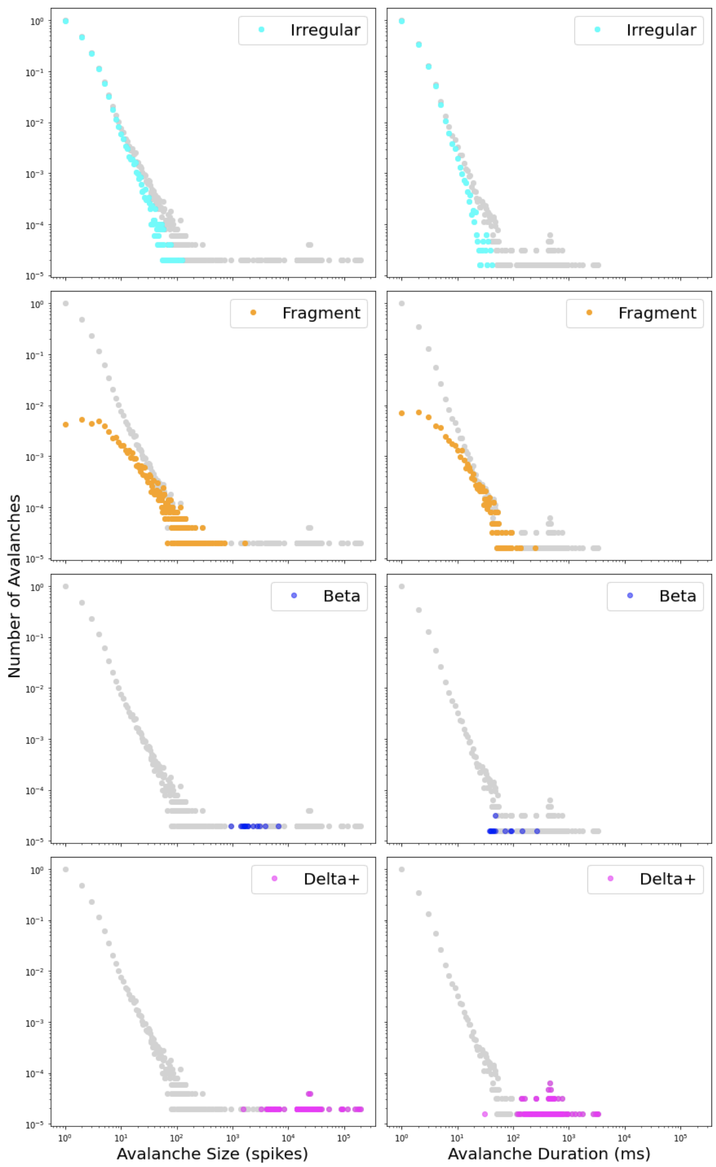

Figure S11. Log-log probability density distributions of avalanche size (first column) and duration (second column) for all avalanches (gray) and each of four avalanche types (sM1_11-23-2020_05; row 4; Fig S2).

bioRxiv preprint doi: https://doi.org/10.1101/2025.01.13.632866; this version posted January 14, 2025. The copyright holder for this preprint (which was not certified by peer review) is the author/funder, who has granted bioRxiv a license to display the preprint in perpetuity. It is made available under aCC-BY 4.0 International license.

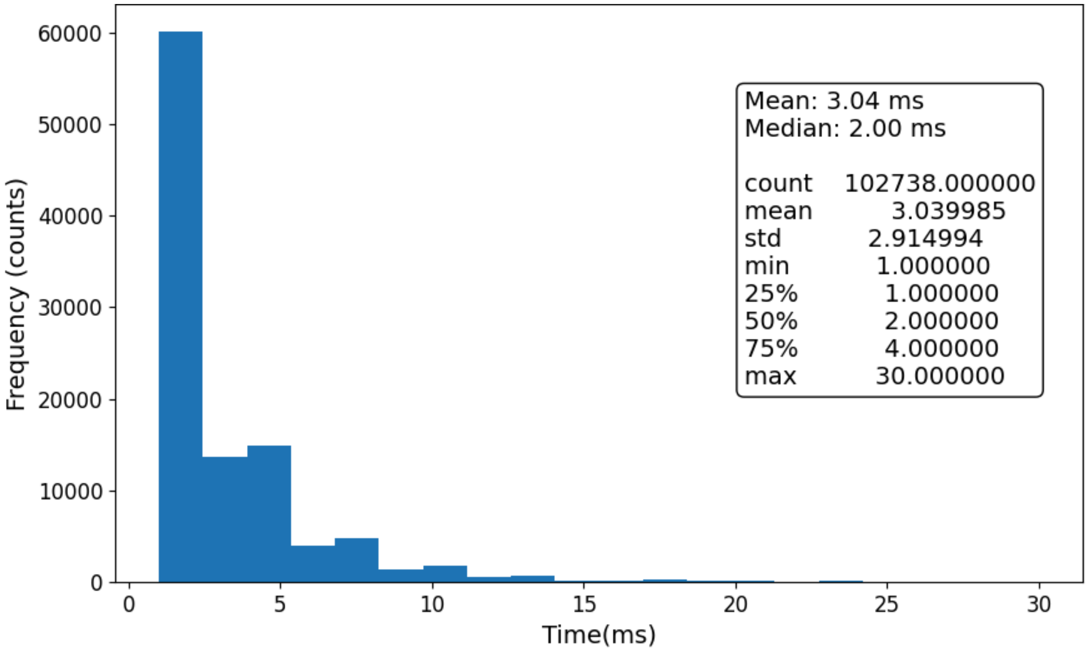

Figure S12. Inter-avalanche intervals in milliseconds (sM1_11-23-2020_05; row 4; Fig S2).

Avalanche Type	Number	Percent Total Num	Size Range (spikes)	Size Range (neurons)	Duration Range (ms)	Total Duration (ms)	Percent Total Dur
Irregular	35,240	92.0%	1-131	1-123	1-46	56,815	12.8%
Fragment	2,717	7.1%	1-1,106	1-1,043	1-179	35,211	8.0%
Beta	66	0.2%	724-39,533	658-5,014	33-1,151	5,437	1.2%
Delta+	283	0.7%	4,156-376,839	1,269-7,684	130-7,131	344,794	78.0%
Total	38,306	100.0%				442,257	100.0%

Table S5. Data from a 10 minutes simulation of M1 (sM1_11–26-2020_01). Row 5 in Fig S2.

bioRxiv preprint doi: https://doi.org/10.1101/2025.01.13.632866; this version posted January 14, 2025. The copyright holder for this preprint (which was not certified by peer review) is the author/funder, who has granted bioRxiv a license to display the preprint in perpetuity. It is made available under aCC-BY 4.0 International license.

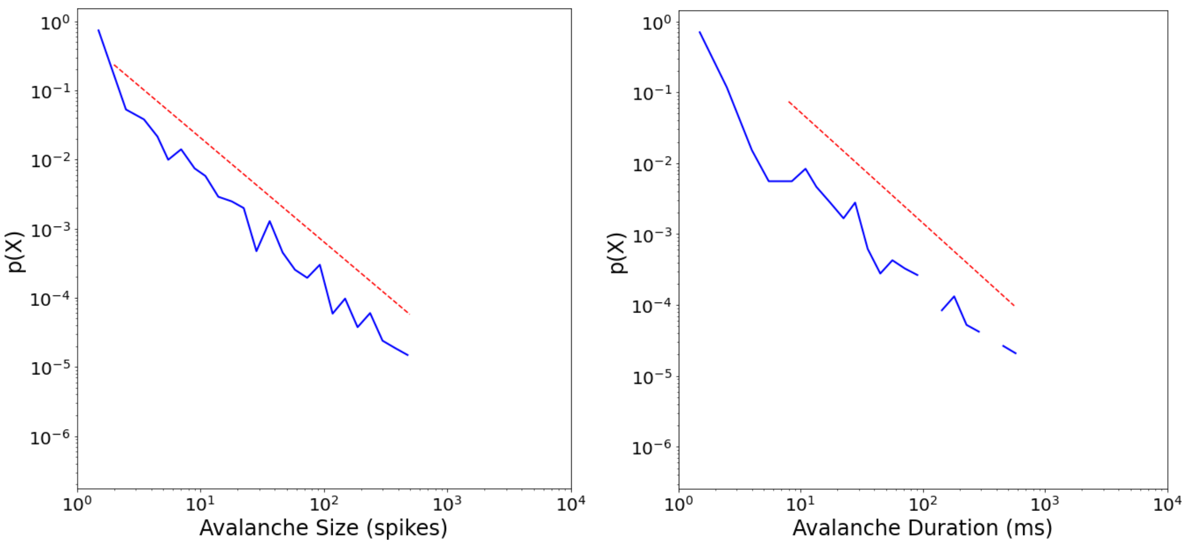

Figure S13. Power-law fits for avalanche size to all neurons in our 10 minute simulation of an M1 cortical column (sM1_11–26-2020_01). Left: Avalanche size probability density distribution with the number of avalanches normalized (y-axis) and the size of the avalanche (number of spikes; x-axis). Power-law fit equals -1.51 (red dashed line; sigma = 0.041, D = 0.045). Right: Avalanche duration probability density distribution showing the number of avalanches normalized (y-axis) and their durations (milliseconds; x-axis). Power-law fit equals -1.56 (red dashed line; sigma = 0.083, D = 0.057). Analyzed using the Python powerlaw package [44].

bioRxiv preprint doi: https://doi.org/10.1101/2025.01.13.632866; this version posted January 14, 2025. The copyright holder for this preprint (which was not certified by peer review) is the author/funder, who has granted bioRxiv a license to display the preprint in perpetuity. It is made available under aCC-BY 4.0 International license.

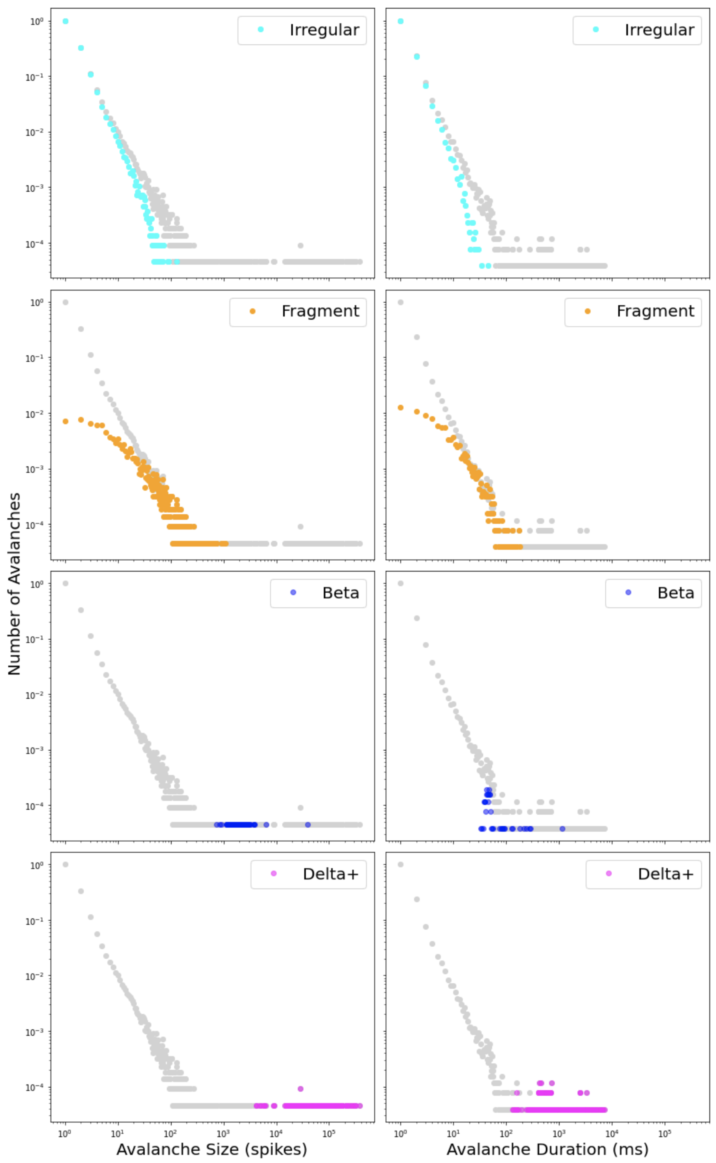

Figure S14. Log-log probability density distributions of avalanche size (first column) and duration (second column) for all avalanches (gray) and each of four avalanche types (sM1_11–26-2020_01; row 5; Fig S2).

bioRxiv preprint doi: https://doi.org/10.1101/2025.01.13.632866; this version posted January 14, 2025. The copyright holder for this preprint (which was not certified by peer review) is the author/funder, who has granted bioRxiv a license to display the preprint in perpetuity. It is made available under aCC-BY 4.0 International license.

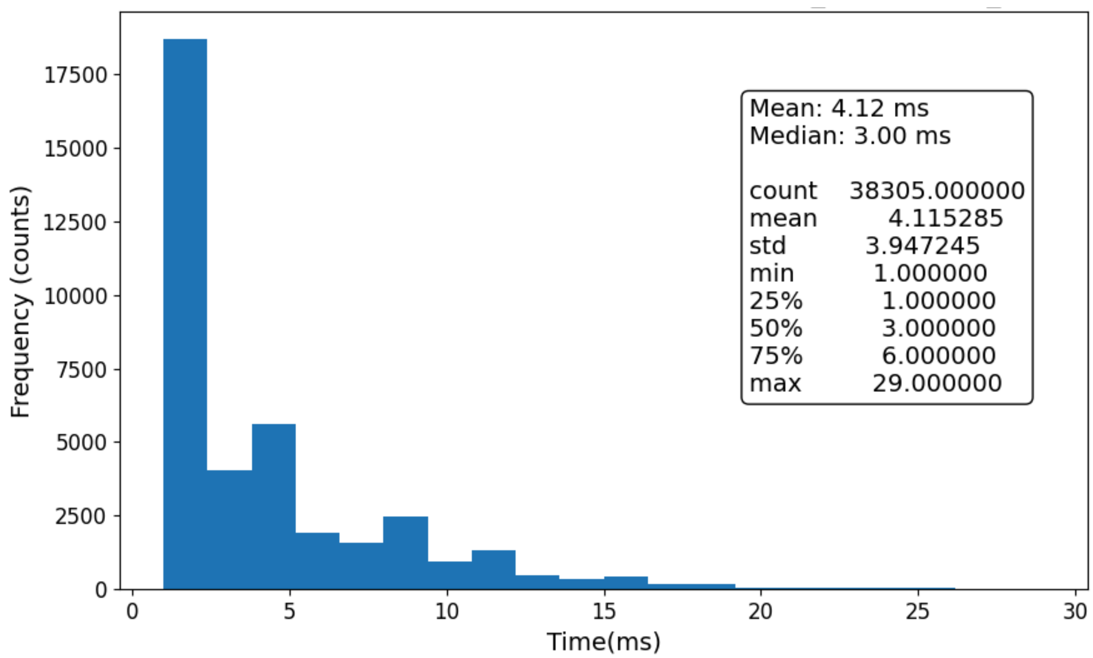

Figure S15. Inter-avalanche intervals in milliseconds (sM1_11–26-2020_01; row 5; Fig S2).

Avalanche Type	Number	Percent Total Num	Size Range (spikes)	Size Range (neurons)	Duration Range (ms)	Total Duration (ms)	Percent Total Dur
Irregular	774	38.0%	1-64	1-64	1-25	2599	0.4%
Fragment	613	30.0%	1-1,102	1-1,064	1-198	8615	1.4%
Beta	259	12.7%	554-5,692	492-4,338	31-310	15,218	2.6%
Delta+	393	19.3%	4,335-352,729	1,307-7,719	101-8,681	570,323	95.6%
Total	2,039	100.0%				596,755	100.0%

Table S6. Data from a 10 minutes simulation of M1 (sM1_11–28-2020_05). Row 6 in Fig S2.

bioRxiv preprint doi: https://doi.org/10.1101/2025.01.13.632866; this version posted January 14, 2025. The copyright holder for this preprint (which was not certified by peer review) is the author/funder, who has granted bioRxiv a license to display the preprint in perpetuity. It is made available under aCC-BY 4.0 International license.

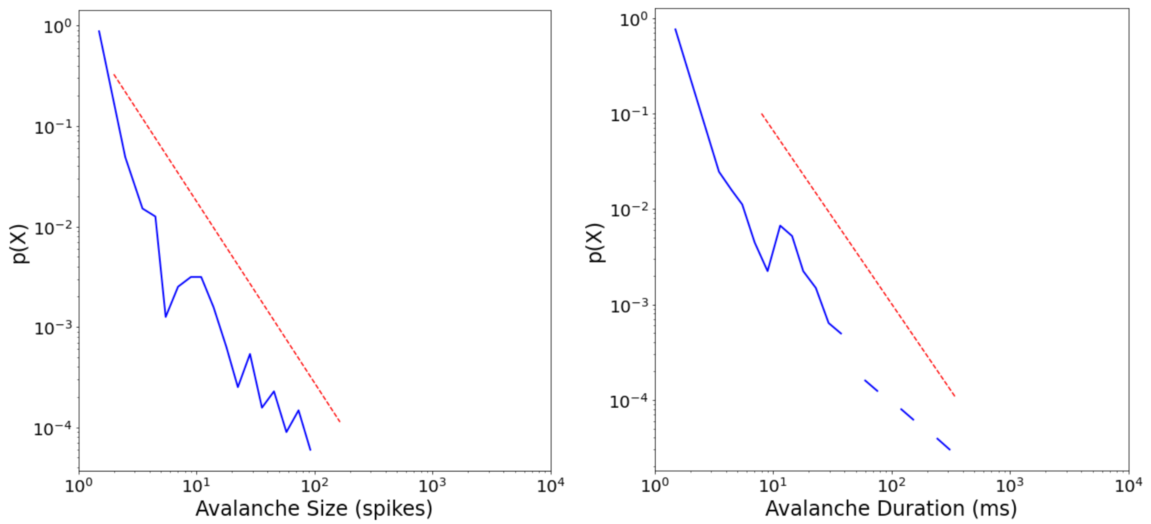

Figure S16. Power-law fits for avalanche size to all neurons in our 10 minute simulation of an M1 cortical column (sM1_11–28-2020_05). Left: Avalanche size probability density distribution with the number of avalanches normalized (y-axis) and the size of the avalanche (number of spikes; x-axis). Power-law fit equals -1.81 (red dashed line; sigma = 0.082, D = 0.075). Right: Avalanche duration probability density distribution showing the number of avalanches normalized (y-axis) and their durations (milliseconds; x-axis). Power-law fit equals -1.82 (red dashed line; sigma = 0.14, D = 0.13). Analyzed using the Python powerlaw package [44].

bioRxiv preprint doi: https://doi.org/10.1101/2025.01.13.632866; this version posted January 14, 2025. The copyright holder for this preprint (which was not certified by peer review) is the author/funder, who has granted bioRxiv a license to display the preprint in perpetuity. It is made available under aCC-BY 4.0 International license.

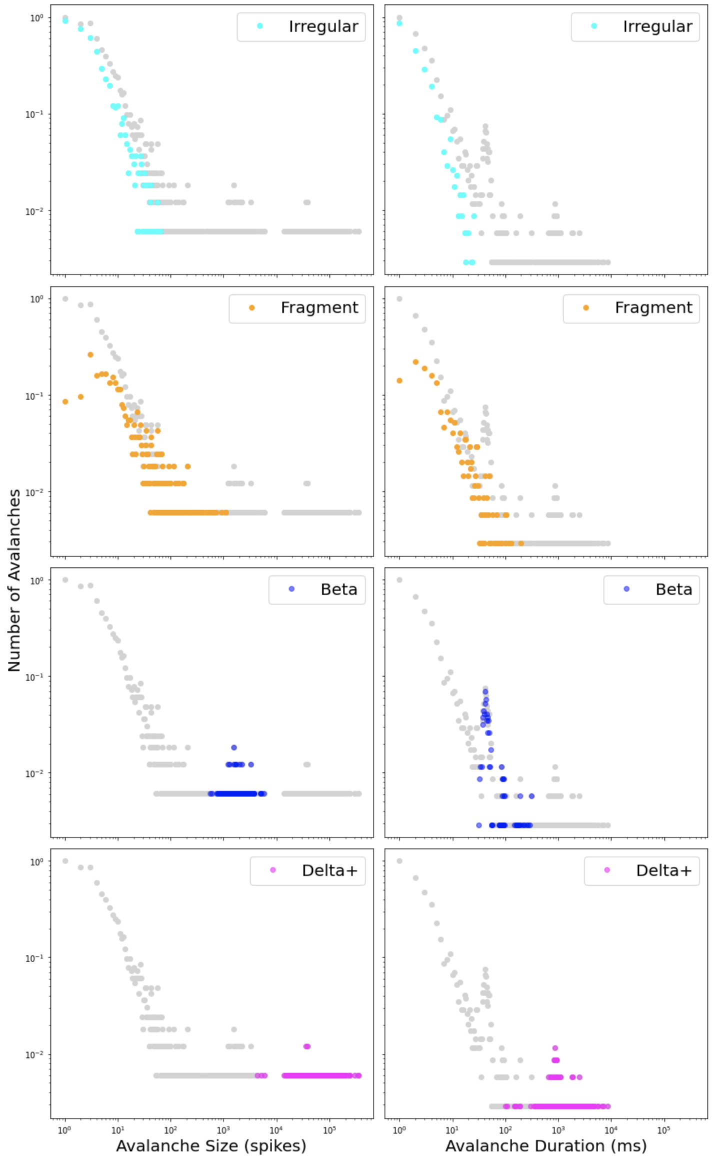

Figure S17. Log-log probability density distributions of avalanche size (first column) and duration (second column) for all avalanches (gray) and each of four avalanche types (sM1_11–28-2020_05; row 6; Fig S2).

bioRxiv preprint doi: https://doi.org/10.1101/2025.01.13.632866; this version posted January 14, 2025. The copyright holder for this preprint (which was not certified by peer review) is the author/funder, who has granted bioRxiv a license to display the preprint in perpetuity. It is made available under aCC-BY 4.0 International license.

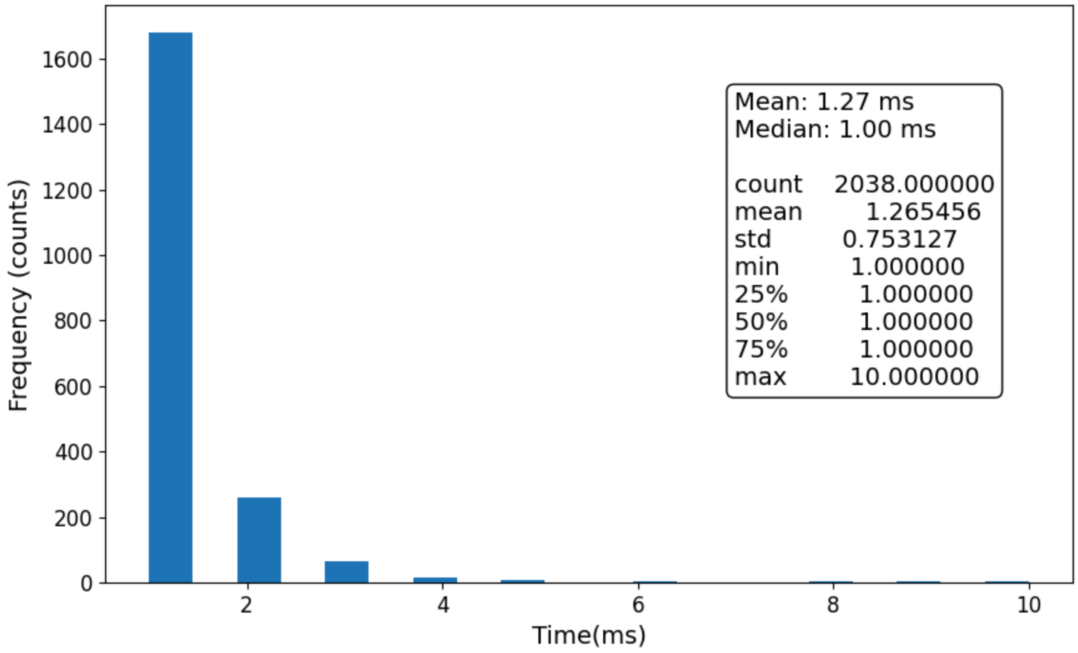

Figure S18. Inter-avalanche intervals in milliseconds (sM1_11–28-2020_05; row 6; Fig S2).

Avalanche Type	Number	Percent Total Num	Size Range (spikes)	Size Range (neurons)	Duration Range (ms)	Total Duration (ms)	Percent Total Dur
Irregular	32,963	100.0%				32963	100.0%
Fragment	0	0.0%					
Beta	0	0.0%					
Delta+	0	0.0%					
Total	32,963	100.0%				32963	100.0%

Table S7. Data from a 10 minutes simulation of M1 (sM1_04–27-2021_17). Row 7 in Fig S2.

bioRxiv preprint doi: https://doi.org/10.1101/2025.01.13.632866; this version posted January 14, 2025. The copyright holder for this preprint (which was not certified by peer review) is the author/funder, who has granted bioRxiv a license to display the preprint in perpetuity. It is made available under aCC-BY 4.0 International license.

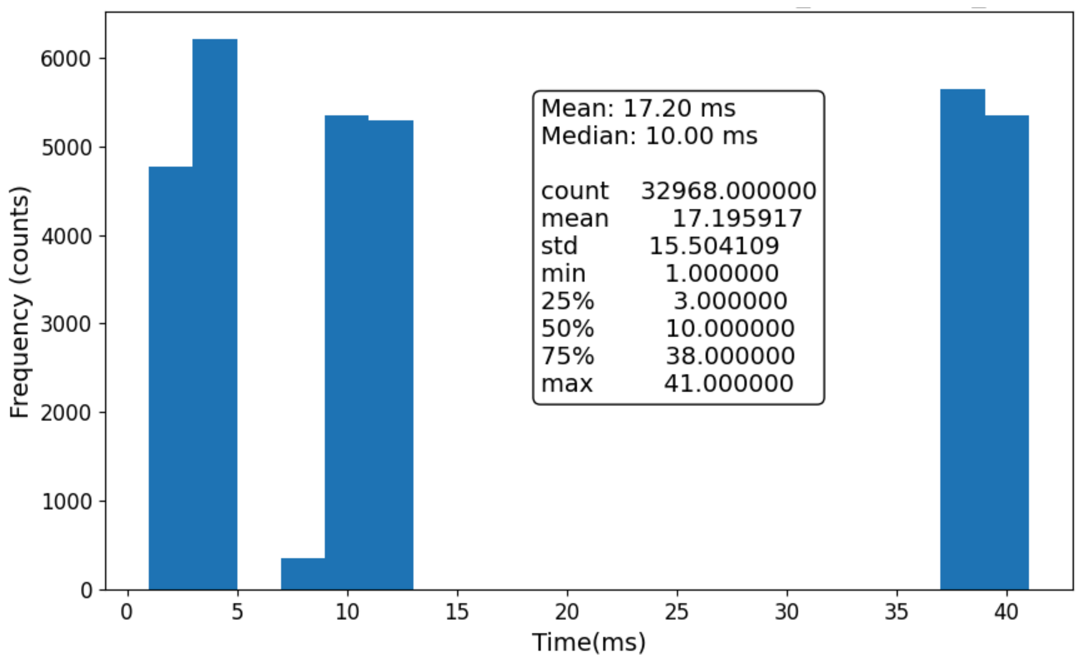

Figure S19. Inter-avalanche intervals in milliseconds (sM1_04–27-2021_17; row 7; Fig S2).
